# A Bibliometric Analysis of Physical Literacy Studies in Relation to Health of Children and Adolescents

**DOI:** 10.3390/children10040660

**Published:** 2023-03-30

**Authors:** Javier Urbano-Mairena, Antonio Castillo-Paredes, Laura Muñoz-Bermejo, Ángel Denche-Zamorano, Jorge Rojo-Ramos, Raquel Pastor-Cisneros, María Mendoza-Muñoz

**Affiliations:** 1 Promoting a Healthy Society Research Group (PHeSO), Faculty of Sport Sciences, University of Extremadura, 10003 Caceres, Spain; 2 Grupo AFySE, Investigación en Actividad Física y Salud Escolar, Escuela de Pedagogía en Educación Física, Facultad de Educación, Universidad de Las Américas, Santiago 8370040, Chile; 3 Social Impact and Innovation in Health (InHEALTH), University Centre of Mérida, University of Extremadura, 06800 Merida, Spain; 4 Physical Activity for Education, Performance and Health (PAEPH) Research Group, Faculty of Sports Sciences, University of Extremadura, 10004 Caceres, Spain; 5 Comprehensive Health Research Centre (CHRC), University of Évora, 7004-516 Évora, Portugal; 6 Research Group on Physical and Health Literacy and Health-Related Quality of Life (PHYQOL), Faculty of Sport Sciences, University of Extremadura, 10003 Caceres, Spain

**Keywords:** bibliometric, healthy behavior, monitoring, physical activity

## Abstract

Regular physical activity (PA) is an essential component of maintaining good health, thereby improving the physical and psychological well-being of the population. PA performed during childhood and adolescence can have repercussions in adulthood, contributing to the prevention of chronic activities and improving quality of life. Given its high relationship with PA, physical literacy could play a crucial role in valuing and participating in a physically active lifestyle, thus addressing low rates of PA participation from an early age. This bibliometric analysis provides a globalized view of physical literacy (PL) and its relationship with health, pathologies, prevention, or intervention among children and adolescents. Publications registered on Web of Science were analyzed using bibliometrics based on data from 141 documents published between 2014 and 2022, while the VOSviewer software v. 1.6.18. was used for the processing and visualization of the data and metadata. The results show an exponential growth in scientific research over the last 8 years, with an accumulation of documents in four journals and a distribution of publications spanning thirty-seven countries and regions. The network of researchers consists of 500 researchers, with the largest number of publications corresponding to 18 co-authors with at least 5 publications. The principal purpose of this research was to identify the most prolific co-authors, most-cited journals and co-authors, and the most relevant keywords.

## 1. Introduction

Nowadays, physical activity (PA) should contribute directly to the primary and secondary prevention of various chronic diseases, such as diabetes, cancer, obesity, overweight, hypertension, and joint diseases [1,2], and scientific evidence has shown that regular PA improves mental health [3], quality of life [4], and well-being [5]. Therefore, PA is an essential component of the maintenance of good health [6].

The combination of the maintenance of physical activity and a healthy lifestyle have always been considered an effective pharmacological strategy for health promotion among all age groups [7,8]. In this regard, the benefits of practicing PA have been extensively scientifically proven. There are also benefits of PA during adulthood. Some of the benefits of exercise during childhood include positive changes in adiposity, superior skeletal and psychological health, and improved cardiorespiratory fitness [9]. The development of motor skills, which also has positive effects during childhood, continues into adulthood [9]. As a general rule, increased PA results in a further improvement in one’s health status [10]. The effects of engaging in PA from childhood and adolescence into adulthood may suggest that regular PA may be of great importance for the prevention of chronic diseases during adulthood [11]. Conversely, low levels of PA and the risk of obesity and overweight are associated with lower life expectancy and a lower quality of life in adulthood [12].

However, despite the reported benefits of PA, the level of engagement in sedentary behaviors, i.e., those involving sitting and low energy expenditure [13], is increasing in the worldwide population. Currently, young populations are increasingly sedentary, resulting in less time spent engaging in daily PA [14]. It is estimated that four out of five adolescents aged 13–15 years do not meet the current minimum recommendations for daily PA [15]. Considering the World Health Organization’s (WHO) recommendations regarding PA, young people should engage in 60 min of moderate and vigorous PA per day, yet more than 80% of adolescents, mostly in EU member states, do not meet these recommendations [16].

Diseases related to sedentary lifestyles and physical inactivity, such as obesity, are reaching epidemic levels in developed countries [14]. From 1975 to 2016, the number of young people between 5–19 years of age with obesity or who were overweight had more than quadrupled (from 4% to 18%) globally [17]. The fact that 6-year-old children present the highest rates of obesity and overweight justifies the interest in implementing participatory programs aimed to promote obesity intervention and health promotion from an early age [18].

This constitutes a strong wake-up call for health professionals and public administration to recognize the magnitude of these problems [19] and participate in the development of administrative strategies and the utilization of public health resources for these issues’ prevention [19,20]. Several countries are already developing programs or initiatives with the aim of preventing pathologies associated with sedentary lifestyles, for which PA is a key focus [21,22,23,24]. Partnerships are even being developed between countries such as the USA, Mexico, and Canada to combat childhood obesity by exchanging data and proposing initiatives in an attempt to improve prevention programs and health promotion in their respective countries [25]. Therefore, it is essential to develop PA programs for children and adolescents worldwide to prevent sedentary lifestyles [26] and chronic diseases [10], thus helping people develop an active lifestyle and reducing sedentary behaviors among youth [4].

In this sense, as the Bulletin of the International Council of Sport Science and Physical Education of the United Nations Educational, Scientific, and Cultural Organization states, physical literacy could play an important role in supporting the motivation, confidence, physical competence, knowledge, and understanding necessary for valuing and participating in a physically active lifestyle [27]. Accordingly, a recent study [28] found that participation in PA corresponded with the level of PL. In order to address the globally low rates of participation in PA from an early age, it would be interesting if future efforts to promote PA focused on motor competence in combination with key psychological constructs (such as confidence, enjoyment, and motivation). As Cairney et al. [29] mentioned, the significance of PL as a determinant of health has been neglected, although this is not a new idea. Very few studies have investigated the association between PL and health. In this regard, it has been proven that a better PL is related to different health parameters, such as body composition [30], physical fitness, blood pressure, and quality of life [31].

Therefore, recently, several research groups have been developing [24] or carrying out programs and/or interventions for the improvement of PL among children and adolescents both during the school day [32,33,34] and out-of-school periods [35,36] as well as among other populations such as university students [37] or adults [38].

Thus, due to the currently growing interest in PL [39] and the importance of the topic in question, this study aimed to assess the current scientific literature on PL in relation to health, pathologies, and preventive programs or interventions performed among children and adolescents to provide the scientific community with a comprehensive and practical overview in an updated and thorough way via a bibliometric analysis of data and metadata from pre-existing specialized publications. Thus, the main objective of this study was to analyze the trends of annual publications in order to identify the most prolific and most-cited journals and co-authors and highlight the most-used keywords and the most relevant articles.

## 2. Materials and Methods

### 2.1. Design and Data

A descriptive bibliometric analysis was carried out to map scientific research published in journals indexed in Web of Science (WoS, developed by Clarivate Analytics, Beijing, China) on PL in relation to health, pathologies, and prevention or intervention among young populations. WoS is one of the most widely used sources for bibliometric analysis by researchers due to the large number of journals indexed on the site and the complete information provided on publications (e.g., regarding co-authors, titles, abstracts, keywords, sources, publishers, citations, etc.), which is used for bibliometric studies published on different topics in numerous journals from different publishers [40,41,42,43]. On 30 November, a search was carried out in the Core Collection database of WoS with the following search vectors: ti = (“physical literacy”) and (ts = (“health*”) or ts = (“pathol*”) or ts = (“prevention”) or ts = (“intervention*”) and (ts = (“child*”) or ts = (“adolesc*”) or ts = (“youth”) or ts = (“young”). Additionally, the search was limited to certain editions, namely, Science Citation Index Expanded (SCI-Expanded), Social Sciences Citation Index (SSCI), and Emerging Sources Citation Index (ESCI), and to certain document types, namely, articles and article reviews. WoS was searched using the following tags: TI (Title Search) and TS (Abstract Search). Data were retrieved from the WoS database in xslx and plain text format and subsequently analyzed in Microsoft Excel for Microsoft 365 MSO version 2206 (Washington, DC, USA) and VoSViewer software v. 1.6.18 (Leiden, The Netherlands).

### 2.2. Data Analysis

While analyzing the data, we assessed the following aspects: (1) exponential growth of science, or Price’s Law, which was assessed through the degree of fit with respect to an exponential ratio of the annual growth of publications [44,45]; (2) concentration of publications in journals, or Bradford’s Law, for which the journals were distributed into thirds according to the number of cited documents, establishing those that comprised at least 33% of the total number of publications as core journals in the subject area, while citations were processed similarly [46,47]; (3) concentration of publications in terms of co-authors, or Lotka’s Law, which states that in any field of knowledge, most articles originate from a small proportion of prolific co-authors who, when identified, can be studied in isolation [48], for which the Hirsch index (h-index) is applied to the documents (“h” articles cited at least “h” or more times) and the prominent co-authors are considered to be prolific co-authors with a document numbering among the most-cited articles; (4) concentration of citations in the articles, or Hirsch index (h-index), which considers “h” articles cited at least “h” times or more [49]; and (5) concentration of keywords, or Zipf’s Law, highlighting the most-used keywords in the set of articles [45].

In addition, VOSviewer software v. 1.6.18 was used for the processing and visualization of the dataset and to determine co-occurrence, for which a fragmentation analysis with clustered visualization outputs was performed [50,51].

## 3. Results

### 3.1. Annual Publications Trend

**Figure 1:**
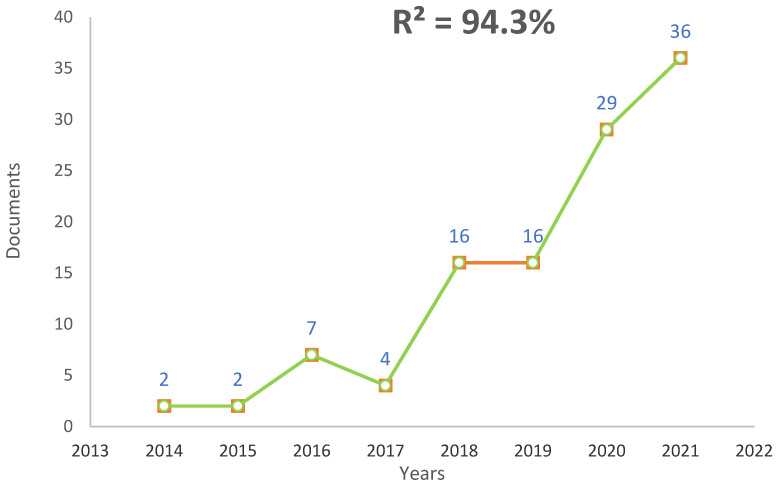
Annual publication trends regarding physical literacy.

We found 141 documents (127 articles and 14 reviews), of which 3 were discarded due to inclusion criteria. These papers were published between 2014 and 2022, with 2021 being the year with the most publications so far, containing a total of 36 publications. This topic has shown exponential growth since 2014 (Figure 1), with a 94.3% (R^2^) fit and an exponential growth rate.

### 3.2. WoS Categories

On WoS, all the documents were classified into 25 different categories, among which Public Environmental Occupational Health was the category with the most articles (43), followed by *Sport Sciences* (41), *Education Educational Research* (39), and *Environmental Science* (14). Table 1 shows, in addition to the categories with the highest number of papers, the journals and publishers with the most publications in each category. The journal with the highest number of papers was *BMC Public Health*, presenting 20 papers, while the publisher with the highest number of publications was Springer Nature (29).

### 3.3. Publications Titles

The documents were issued in a total of 65 journals. The core of the Bradford journals estimated, according to the number of publications in each journal, comprised four journals, all of them with at least six published papers, accounting for 33.33% of the total number of publications. The journal with the most published papers was “*BMC Public Health*” in Q2, accounting for 13.77% of all papers (19). The next journal with the most published papers was “*International Journal of Environmental Research and Public Health*” in category Q1, accounting for 10.14% of all publications (14). The distribution followed by Bradford’s core (four journals), Zone I (19) and Zone II (42), according to the number of papers published by the journals, was under Bradford’s theoretical series.

In terms of number of citations (Table 2), the journal with the highest number of citations was “*BMC Public Health*” (437), representing 27.08% of the total number of citations. The journal with the second highest number of citations was “*Sports Medicine*” in category Q1, with 247 citations, amounting to 15.30% of the total number of citations. Finally, the third most-cited journal, with 102 citations (6.32% of the total), was “*Journal of Teaching in Physical Education*” in category Q2.

### 3.4. Prolific and Influential Co-Authors

**Figure 2:**
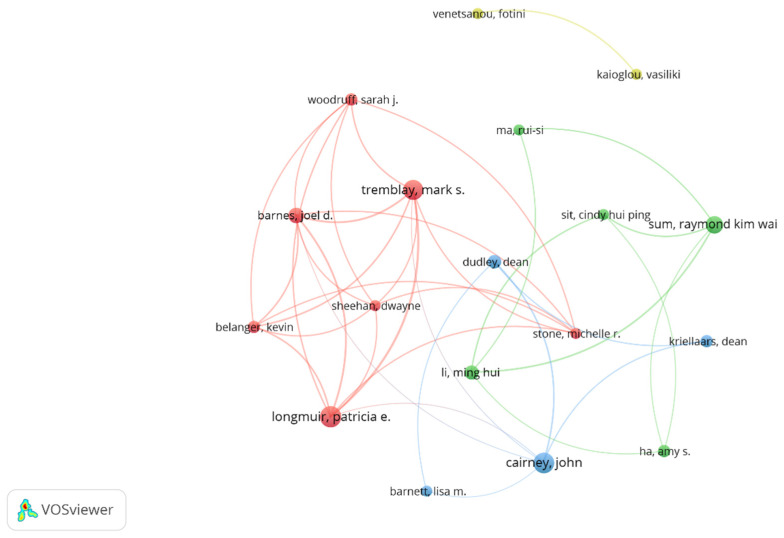
Prolific co-authors.

Figure 2 shows the list of the prolific co-authors in this subject area. Table 3 shows the number of prolific co-authors, their affiliations, countries of origin, and number of papers and citations. Of the total number of co-authors, the majority (eight) belong to the country with the most publications on the subject, Canada. However, the third most-published country (USA) is not represented in this list. Countries such as Australia (three), the People’s Republic of China (five), and Greece (two) complete this list, ranking among the countries with the highest number of publications.

**Figure 3:**
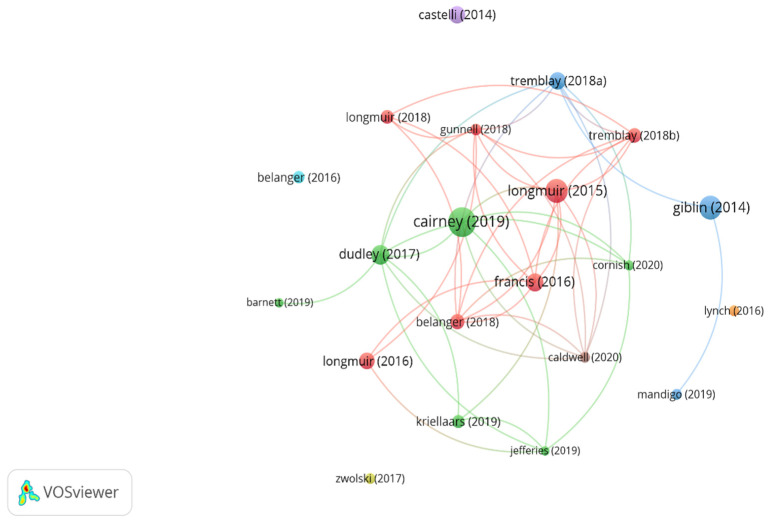
Most-cited articles.

Twenty-one papers were found with twenty-one or more citations (h-index = 21). Figure 3 shows a graph of the articles with the highest number of citations.

When considering the prominent co-authors, i.e., those with more than five papers for which at least one is indexed in the h-21 index, the total number of prominent co-authors decreased from 18 to 11 authors. Table 4 shows the prominent co-authors together with the total number of papers, the total number of papers indexed in the h-21 index, the article with the highest number of citations, and the number of citations of the articles.

### 3.5. Countries/Regions

**Figure 4:**
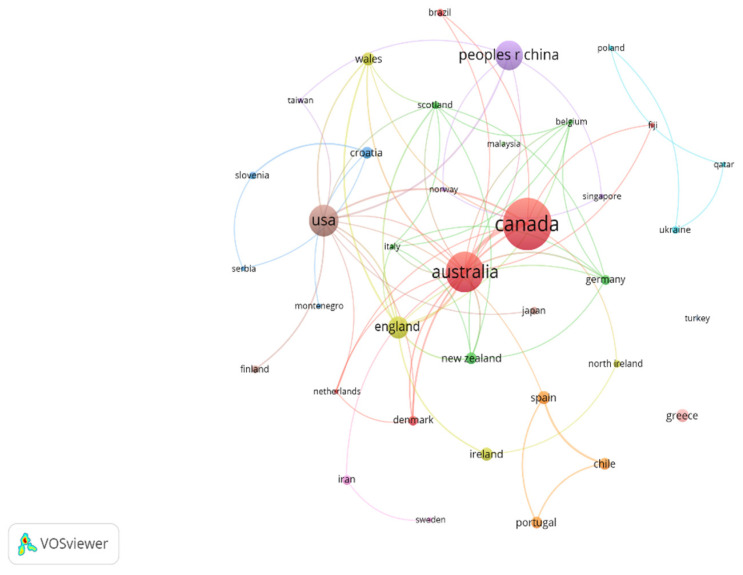
Countries/regions.

The co-authors of the published papers belong to 37 countries/regions with at least one publication (Figure 4). The main countries with the highest number of published papers were Canada with 51 papers and Australia with 33 papers, followed by the USA (22), the People’s Republic of China (20), and England (12). Regarding the number of citations, Canada was the country with the most citations (1040), followed by Australia (483), the USA (395), England (202), and New Zealand (117).

However, the country with the highest number of links that were co-authored was Australia (36), followed by Canada (25), the USA and England with 19 links, and Wales (9). Figure 3 shows the countries/regions with the highest number of co-authored links as well as the clusters formed between them. (Graph: Analysis (Fractionalization), Attraction (9), and Repulsion (−6). Clustering: Resolution (1); Minimum cluster size (1). Node size (Documents); Color (Cluster)).

### 3.6. Author Keywords

**Figure 5:**
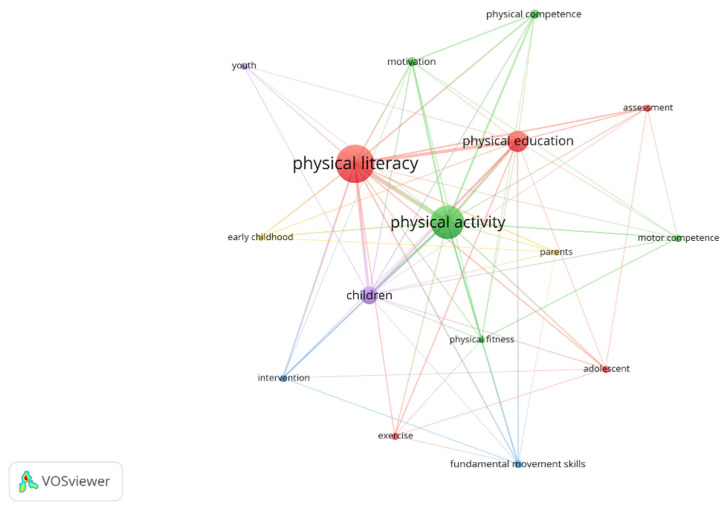
Authors’ keywords.

The authors used a total of 332 keywords in all the documents analyzed (Figure 5). By applying Zipf’s law, it was estimated that the most-used keywords should be a number equal to or less than 18. Accordingly, 16 keywords with 6 or more occurrences were found.

In total, the most-used terms by the authors were as follows: Physical Literacy (66 occurrences), Physical Activity (56 occurrences), Physical Education (30 occurrences), and Children (24 occurrences). Figure 4 shows the prominent author keywords and how they were interrelated in the documents analyzed as well as the clusters formed between them, for which two main clusters were found: red, which was associated with the word Physical Literacy, mainly relating to other keywords such as Physical Education, Assessment, Exercise, or Adolescent, and Green, which was associated with the keyword Physical Activity, relating to keywords such as Physical Competence, Motor Competence, Physical Fitness, or motivation. (Graph: Analysis (Fractionalization), Attraction (8), and Repulsion (−5). Clustering: Resolution (1); Minimum cluster size (1). Node size (Documents); Color (Cluster)).

## 4. Discussion

The present study aimed to assess, in relation to health, pathology, and prevention or intervention measures performed, the trends in the scientific literature on PL among children and adolescents in a comprehensive and updated manner in an attempt to provide a general and practical overview to the scientific community. After examining the bibliometric studies on PL, only one study was found in which PL is analyzed at a general level, including with respect to some general aspects of health [39]. On the other hand, a scoping review that explored PL in the context of health was also found [57], along with a general mapping study where PL was analyzed from different perspectives, depending on the planned objectives [58]. Therefore, this is the first bibliometric study that has attempted to analyze the literature and highlight the most prolific and prominent co-authors, the countries with the most scientific studies, and the most-used keywords in the field of PL and health, pathologies, and prevention or interventions measures carried out among adolescents and schoolchildren.

The first document on the subject of which we are concerned, and for which we have a record, was published in 2014. In this year, two articles were published on the subject. Since then, there has been a considerable increase in the scientific literature on this topic, with the year 2021 constituting the year with the highest number of scientific studies (36 documents). Therefore, it can be surmised that there is a growing interest in this subject (R2: 94.3%). The most relevant document, according to the number of citations, was “Physical Literacy, Physical Activity and Health: Toward an Evidence-Informed Conceptual Model” with 147 citations. However, as highlighted by Cornish, Fox, Fyfe, Koopmans, Pousette, and Pelletier [57], the nature and direction of the relationship between PL, PA, and health requires further exploration in order to clarify the role of PL as a catalyst for promoting PA, decreasing disease burden, and bettering health and well-being [57].

One of the most important findings to note in this document is the evident relevance to the scientific community of the acquisition of reliable and validated assessment instruments—and in a unified way—of PL both in the field of PA and in physical education (red cluster; Figure 5). Such instruments allow for the monitoring and control of PL along with the associated health benefits. Recent studies have shown that among children and adolescents, there is an association between PL and different health parameters, such as body composition [30,31], physical fitness, blood pressure, and health-related quality of life [31]. En este sentido, dos de los tres documentos más citados sobre la AF (Table 4), tratan sobre la evaluación. Entre los instrumentos de evaluación más utilizados encontramos CAPL-2 [52] or Play Tools [59]. In addition, different countries have tried to develop different country-specific assessment models [60]. Thus, there is growing interest and research in developing comprehensive instruments that can comprehensively assess PL.

Concerning the authors’ keywords, two primary clusters were observed: “Physical Literacy” and “Physical Activity”. In addition, other words appear in both clusters, such as “motor competence”, “children”, “motivation”, “children”, and/or “Assessment”. This is in line with the results presented in [58], wherein the two main clusters were in line with those of the present paper. This was also analyzed in [39], in which similar results were obtained. All this may lead us to the idea that when we refer to PL, it is mostly with respect to the educational sphere and concerning school-age children.

Canada can be considered one of the greatest drivers of PL worldwide; accordingly, it is the country with the greatest body of research both on the topic addressed in this study and on PL in general [39]. This is because in Canada, most PL-related sectors (sports, recreation, PA, education, and public health) have embraced PL and are making it a core priority [56]. Proof of this lies in the multitude of tools that have originated in this country (Passport for Life [61]; 60MKC [62]; CAPL-2 [52]; or Play Tools [59]). The development of LP programs and resources has enabled the establishment of new partnerships between sectors in the country. Regarding the interventions that have been carried out regarding PL among children and adolescents, most of them focus on some of the domains that constitute PL and not the development of PL as a whole, in addition to being carried out within the school environment either during break periods [34], physical education classes [32,33], or in out-of-school settings either with the children themselves [35,36] or as assessed by their parents [63], and only a few of these interventions assess health-related variables [24]. As highlighted [60,64], it is difficult to make a conclusive statement regarding the effectiveness of an intervention program due to the paucity of intervention studies on PL promotion. However, due to the previously demonstrated link between PL and health, it is especially necessary to develop interventions based on PL that seek to improve health-related variables and thus prevent different pathologies, including obesity, given its direct relationship with body composition [30,31].

In this sense, the monitoring of PL will facilitate the detection of the deficiencies that may exist in each domain in each population and, therefore, guide appropriate interventions, thereby rendering them more effective. As highlighted in [65], children with various chronic medical conditions are equal to their healthy peers, but children without medical conditions are more physically competent than children with medical conditions, although the latter are more motivated and confident, so programs based on physical competence interventions (motor skills, physical fitness, etc.) rather than motivation or education could be more useful.

One of the strengths of this study is that it provides an overview of PL and health, pathologies, and prevention and intervention measures that have recently been carried out among child and adolescent populations, specifying the current lines of research and compiling relevant information. All this constitutes an attempt to highlight the importance of PL to the health of this population because PL could play an important role; for example, a physically literate child can move with skill and confidence in highly physically demanding situations, understand the physical environment, anticipate the needs of movement, and respond intelligently and imaginatively to the difficulties he/she faces [27]. On the contrary, those who do not have a high level of PL will try to avoid PA whenever possible; thus, their level of confidence in their physical ability will be low, and they will not be motivated to participate in structured PA [56], with all the health-related biases for which physical inactivity is already considered a pandemic [66]. Thus, physical inactivity in childhood is a global health problem and a persistent major public health problem that has been worsened by COVID-19 [67]. In this regard, the present findings lend support to the growing movement to consider PL as a multidimensional concept in order to understand and address this problem [57].

In short, developing and improving PL could be crucial for improving physical-activity-related habits in the future. Advancing PL could lead to an improvement in all four domains (motivation and confidence, daily activity, knowledge, and understanding and physical competence), thus improving children’s physical competence and enabling them to collaborate in physical activities more easily and safely, which, in turn, increases their confidence and self-esteem, makes them feel more confident and self-assured, and, therefore, results in a greater interest in physical activity and a more positive attitude towards sport and exercise. Furthermore, children who are physically literate are more likely to participate in physical activity on a regular basis, which can lead to a more active and healthier lifestyle in general, which, in turn, will lead to improved cardiovascular and muscular health and help prevent diseases such as obesity. Therefore, it is vital to encourage physical literacy among children from an early age to help them develop physical skills, self-confidence, and an active and healthy lifestyle.

One of the limitations we can highlight is that this study has focused on PL from a general health point of view, so a future line of research could include the bibliometric analysis of PL and health in different contexts such as public health, recreation, sports, and education [68]. It could even be interesting to study it in combination with other literacies, such as health literacy, whose linkage has been explored in recent publications [69,70]. This would include the analysis of more specialized documents and the increase in the amount of relevant information on the relevant topic. The incompatibility of using different sets of databases, in comparative terms, would be another frequent limitation of mainly bibliometrics, mainly in comparison with the impact of their different coverage of journals, proceedings, and books, which, in this study, forced us to limit ourselves to a specific set of databases (in this case, WoS) in order to perform an analysis on a broader coverage of data fields and metadata [71,72,73,74].

Therefore, based on these results, as future lines of research, it would be interesting to evaluate PL among children and adolescents and monitor its evolution from childhood to adulthood. In addition, this study can serve as a starting point from which to encourage researchers to carry out an analysis based on the improvement of PL and, consequently, health as well as the study of its relationship with other important parameters in the child and adolescent populations that are related directly or indirectly with health such as physical and mental health, socioeconomic factors, or environmental factors.

## 5. Conclusions

This bibliometric study aimed to evaluate the trends of the existing scientific literature on PL in relation to health, pathologies, and prevention or intervention measures carried out among children and adolescents. Since 2014, the year the first paper was published on this topic, there has been an exponential increase in publications to date, demonstrating the increasing interest in this topic among the scientific community. Regarding the origin of the publications, the papers were published in a total of 64 journals. *BMC Public Health*, *International Journal of Environmental Research and Public Health*, *Physical Education and Sport Pedagogy*, and the *Journal of Teaching in Physical Education* were the journals with the most articles published, constituting at least six papers and 33.33% of the total number of publications.

In reference to the co-authors, 18 co-authors were prolific co-authors with more than five published articles. In addition, 11 of these 18 co-authors were considered prominent, having more than 5 articles and with at least one of them in the h-21 index territory. Canada (24.17%) was the country with the highest number of publications, including eight co-authors among the prolific authors.

The articles concentrate the topics into two main clusters, one related to PL and the other to PA. These two highest-mentioned concepts are the most-used keywords (66 and 56 occurrences, respectively), in addition to physical education (30) and children (24).

Therefore, it is clear that PL is a growing and vitally important topic, especially in the child and youth populations, as the development and improvement of PL could be crucial for improving PA habits in the future. Developing PA could lead to an improvement in all four domains (motivation and confidence, daily activity, knowledge, and understanding and physical competence), thereby enabling children to participate in physical activities more easily and safely and increasing their confidence and self-esteem and thus allowing them to participate in physical activities on a regular basis, which can lead to a more active and healthy lifestyle in general, and all this in addition to the respective health benefits that come with regular engagement in PA. Therefore, it is vital to encourage PL among children from an early age to help them develop physical skills, self-confidence, and an active and healthy lifestyle.

## Figures and Tables

**Table 1:** WoS categories.

WoS Categories	Doc.	Prolific Journals	Doc.	Prolific Publishers	Doc.
Public Environmental Occupational Health	43	*BMC Public Health*	20	Springer Nature	29
Sport Sciences	41	*International Journal of Environmental Research and Public Health*	14	MDPI	24
Education Educational Research	39	*Physical Education and Sport Pedagogy*	7	Taylor & Francis	23
Environmental Sciences	14	*Journal of Teaching in Physical Education*	6	Elsevier	11
Hospitality Leisure Sport Tourism	13	*European Physical Education Review*	5	Human Kinetics Publ Inc	10

Doc. (Number of documents).

**Table 2:** Prolific journals.

Bradford’s Zone	Journals	Doc.	%Doc.	Cit. *	JIF	Q.	%O.A	Cumulative Frequency
CORE	*BMC Public Health*	19	13.77%	437	4.135	Q2	99.59%	13.77%
	*International Journal of Environmental* *Research and Public Health*	14	10.14%	84	4.614	Q1	96.11%	23.91%
	*Physical Education and Sport Pedagogy*	7	5.07%	28	4.638	Q1	19.16%	28.99%
	*Journal of Teaching in Physical Education*	6	4.35%	102	2.66	Q2	1.74%	33.33%
ZONE 1	*European Physical Education Review*	5	3.62%	38	3.675	Q1	16.67%	3.62%
	*Applied Physiology Nutrition and Metabolism*	4	2.90%	22	3.016	Q2	7.89%	6.52%
	*Biology-Basel*	3	2.17%	9	5.168	Q1	94.78%	8.70%
	*Children-Basel*	3	2.17%	12	2.835	Q2	95.35%	10.87%
	*Curriculum studies in Health and Physical* *Education*	3	2.17%	15	N/A	N/A	14.29%	13.04%
	*Frontiers in Public Health*	3	2.17%	22	6.461	Q1	99.17%	15.22%
	*Journal of Exercise Science & Fitness*	3	2.17%	28	3.465	Q2	100.00%	17.39%
	*Journal of Physical Activity & Health*	3	2.17%	69	3	Q2	0.64%	19.57%
	*Psychology of Sport and Exercise*	3	2.17%	3	5.118	Q1	7.47%	21.74%
	*Frontiers in Sports and Active Living*	2	1.45%	0	N/A	N/A	98.51%	23.19%
	*Journal of Physical Education Recreation and Dance*	2	1.45%	2	N/A	N/A	0.00%	24.64%
	*Journal of Science and Medicine in Sport*	2	1.45%	8	4.597	Q1	8.42%	26.09%
	*Physical Educator-Us*	2	1.45%	21	N/A	N/A	0.00%	27.54%
	*Quest*	2	1.45%	88	2.891	Q2	4.44%	28.99%
	*Revista de Psicologia del Deporte*	2	1.45%	3	0.936	Q4	0%	30.43%
	*Science of Gymnastics Journal*	2	1.45%	4	N/A	N/A	0%	31.88%
	*Sport Education and Society*	2	1.45%	5	3.586	Q1	14.92%	33.33%
	*Sports Medicine*	2	1.45%	247	11.928	Q1	31.33%	34.78%
	*Sports Medicine—Open*	2	1.45%	10	6.766	Q1	100%	36.23%

Doc. (number of documents); %Doc (percentage of documents); Cit. (number of citations); JIF (journal impact factor); Q. (JIF quartile); %O.A. (percentage of open access articles); N/A: not applicable; *: times cited in WoS Core Collection.

**Table 3:** Prolific co-authors.

Authors	Affiliation	Country/Region	Doc.	Cit.
Longmuir, Patricia E.	University of Ottawa	Canada	16	494
Cairney, John	University of Queensland	Australia	15	403
Tremblay, Mark S.	Children’s Hospital Eastern Ontario Research Institute	Canada	14	479
Sum, Raymond Kim Wai	Chinese University of Hong Kong	China	11	75
Barnes, Joel D.	Children’s Hospital Eastern Ontario Research Institute	Canada	9	284
Li, Ming Hui	Chinese University of Hong Kong	China	8	32
Dudley, Dean	Macquaire University	Australia	7	284
Belanger, Kevin	Children’s Hospital Eastern Ontario Research Institute	Canada	6	145
Ha, Amy S.	Chinese University of Hong Kong	China	6	27
Kriellaars, Dean	University of Manitoba	Canada	6	275
Woodruff, Sarah J.	University of Windsor	Canada	6	153
Barnett, Lisa M.	Deakin University	Australia	5	29
Kaioglou, Vasiliki	National & Kapodistrian University Athens	Greece	5	23
Ma, Rui-Si	Jinan University	China	5	16
Sheehan, Dwayne	Mount Royal Uni, Fac. of Health, Community, and Education	Canada	5	108
Sit, Cindy Hui Ping	Chinese University of Hong Kong	China	5	22
Stone, Michelle R.	Dalhousie University	Canada	5	117
Venetsanou, Fotini	National and Kapodistrian University Athens	Greece	5	23

Doc. (Documents); Cit. (Citations).

**Table 4:** Prominent Co-authors.

Author(s)	Total Documents	Documents in H-Index 21	Most-Cited Document	Times Cited *
Longmuir, Patricia E.	16	7	The Canadian Assessment of Physical Literacy: methods for children in grades 4 to 6 (8 to 12 years) [52]	101
Tremblay, Mark S.	14	8		
Cairney, John	15	5	Physical Literacy, Physical Activity and Health: Toward an Evidence-Informed Conceptual Model [29]	147
Dudley, Dean	7	3		
Kriellaars, Dean	6	4		
Barnes, Joel D.	9	5	The Canadian Assessment of Physical Literacy: Development of a Model of Children’s Capacity for a Healthy, Active Lifestyle Through a Delphi Process [53]	65
Belanger, Kevin	6	3	The relationship between physical literacy scores and adherence to Canadian physical activity and sedentary behaviour guidelines [54]	47
Woodruff, Sarah J.	6	3		
Sheehan, Dwayne	5	2		
Barnett, Lisa M.	5	1	Guidelines for the Selection of Physical Literacy Measures in Physical Education in Australia [55]	21
Stone, Michelle R.	5	1	Physical literacy levels of Canadian children aged 8–12 years: descriptive and normative results from the RBC Learn to Play-CAPL project [56]	44

* Times Cited according to WoS Core Collection (until 30 November 2022).

## Data Availability

Datasets are available through the corresponding author upon reasonable request.
